# Development and Application of a 1,8‐Naphthalimide‐Based Fluorescent Probe for Sensitive Detection of Hydrogen Sulfide in Human Blood Serum

**DOI:** 10.1002/bio.70295

**Published:** 2025-08-19

**Authors:** Aleksandar Széchenyi, Mirela Samardžić, Mateja Budetić, Ines Drenjančević, Nikolina Kolobarić, Gábor Mikle, Barna Kovács, Andrea Dandić

**Affiliations:** ^1^ Department of Chemistry Josip Juraj Strossmayer University of Osijek Osijek Croatia; ^2^ Green Chemistry Research Group, János Szentágothai Research Centre University of Pécs Pécs Hungary; ^3^ Institute of Pharmaceutical Technology and Biopharmacy, Faculty of Pharmacy University of Pécs Pécs Hungary; ^4^ Department of Physiology and Immunology, Faculty of Medicine Osijek J. J. Strossmayer University of Osijek Osijek Croatia; ^5^ Department of General and Inorganic Chemistry, Faculty of Sciences University of Pécs Pécs Hungary; ^6^ Research Group for Selective Chemical Syntheses, HUNREN‐PTE Pécs Hungary

**Keywords:** 1,8‐naphthalimide, azide group, fluorescent probe, human blood serum, hydrogen sulfide

## Abstract

This work implies the preparation, structural and fluorescent characterization, and application of the 1,8‐naphthalimide‐based fluorescent probe for H_2_S detection in human blood serum. The mechanism of fluorescent detection of H_2_S is based on the reduction of the azide group, as the chemical reactive site, in the fluorescent probe structure, to the amino group. Compound **2** possesses another important structural motif, ethanolamine moiety, which modulates physicochemical properties. The synthetic pathway for the preparation of compound **2** is consisted of two reaction steps, and the target compound was prepared with a yield of 56%. The prepared compound **2** was characterized using ^1^H and ^13^C NMR spectroscopy and elemental analysis. During the fluorescence spectra measurements, several influences on fluorescence intensity were investigated, including pH, time dependence, selective response, and influence of H_2_S concentration. The detection limit was calculated, which was 0.16 μmol L^−1^. For examination of the application of compound **2** for H_2_S detection in real samples, compound **2** was successfully applied for H_2_S detection in human blood serum. The concentration of H_2_S in the human serum sample was 16.2 μmol L^−1^.The accuracy of H_2_S determination in human blood serum samples is confirmed by the standard addition method and UV‐Vis spectrophotometry method using methylene blue.

## Graphical abstract

The target 1,8‐napthalimide‐based fluorescent probe was prepared and successfully applied for H_2_S detection in human blood serum whereby the concentration of H_2_S in human blood serum was found to be 16.2 μmol L^−1^. The accuracy of the developed method was confirmed by the standard addition method and UV‐Vis spectrophotometry method using methylene blue.

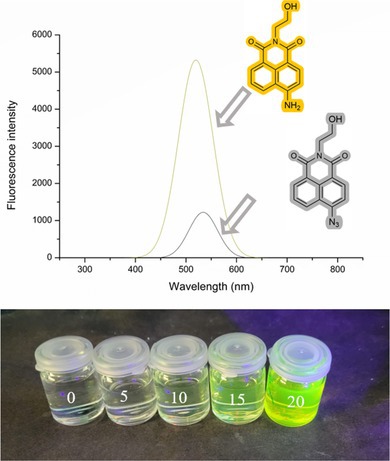

## 1 Introduction

Identifying and detecting small gaseous molecules as signal transmitters in living systems involved in various physiological and pathological processes is of great importance. These molecules are called gasotransmitters and include nitric oxide (NO), carbon monoxide (CO), and hydrogen sulfide (H_2_S) [1]. Hydrogen sulfide is the third gasotransmitter that is endogenously generated by the action of three main enzymes, namely, cystathionine‐β‐synthase (CBS), cystathionine‐γ‐lyase (CSE), and 3‐mercaptopyruvate sulfurtransferase (MST) [2]. In the case of enzyme CBS, H_2_S is produced from cysteine by β‐elimination reaction and by β‐replacement reaction, which implies condensation of cysteine with homocysteine. Enzyme CBS is localized to human chromosome 21 [3] whereby in patients with Down syndrome, CBS levels were found to be 1.5‐fold greater compared to those in healthy individuals. Due to the overproduction of H_2_S in patients with Down syndrome, mental disorders can occur [4]. As CBS is the main H_2_S‐forming enzyme in the central nervous system (CBS), CSE is the main H_2_S‐forming enzyme in the cardiovascular system [5]. The last mentioned enzyme responsible for H_2_S formation, MST, is mainly localized to neurons in the brain and retina [6].

Furthermore, H_2_S is an environmental pollutant, a colorless, flammable, and toxic gas with high toxicity. A concentration of 500 ppm [7] of H_2_S can cause respiratory disorders because the lethal dose of H_2_S is 1000 ppmv [8]. Moreover, H_2_S is involved in immune response, signal transduction, and energy production, whereas endogenous H_2_S plays a role in various physiological functions, including regulating blood pressure, neurotransmission, anti‐inflammatory effects, vasodilation, antioxidation, and apoptosis [9, 10]. According to the literature, the toxicity of H_2_S can be related to respiratory complex activities in mitochondria, which result in cellular incapability to metabolize oxygen in an oxidative manner [11, 12]. A recent study explained the relationship between H_2_S levels, asthma, and chronic obstructive pulmonary disease (COPD) [13].

Although H_2_S has been known as a toxic compound for a long time, according to recent studies, it exhibits an antiapoptotic effect in cancer cells via various mechanisms. One of the mechanisms of action includes reactive oxygen species (ROS) scavenging due to its reducing property [14]. Additionally, ROS in organisms are formed as by‐products of oxygen metabolism and can cause damage to biologically important molecules, although their higher level is related to oxidative stress development. Oxidative stress is a physiological state caused by an imbalance between ROS production and their elimination from the organism [15]. H_2_S possesses both direct and indirect antioxidant capacities. The direct antioxidant capacity of H_2_S is related to its chemical properties, which enable it to neutralize ROS. On the contrary, the indirect antioxidant capacity of H_2_S is related to its regulation of key antioxidant enzymes, including superoxide dismutase (SOD), catalase, and glutathione peroxidase (GPx) [16, 17]. The enzymes mentioned above are crucial for maintaining redox homeostasis and play a role in ROS neutralization [18].

Considering all the physiological functions of H_2_S, its detection has become a theme of great importance. For that purpose, a large number of H_2_S detection methods, including UV–visible (UV‐Vis) spectrophotometry method using methylene blue, electrochemistry, gas chromatography, high‐performance liquid chromatography (HPLC), inductively coupled plasma atomic emission, UV‐Vis absorption spectrometry, and fluorescence spectroscopy, have been developed [19, 20, 21]. Moreover, in the literature, methods are also described, which imply the combination of fluorescence spectroscopy and HPLC in order to detect H_2_S [22, 23, 24]. For example, Lee et al. [24] prepared a fluorescent probe for selective H_2_S detection in serum whereby the product formed after the reaction with H_2_S was confirmed using HPLC. The listed methods possess certain disadvantages; for example, in the case of UV‐Vis spectrophotometry method using methylene blue, the disadvantage is the low detection sensitivity, which is in the micromolar range. Additionally, this method requires a long incubation period, which is not suitable for continuous measurements of H_2_S in physiological conditions [25, 26]. Chromatography‐based methods are incapable of real‐time monitoring in biological samples, especially under hypoxic or anoxic conditions [25]. Electrochemical methods must be highlighted from the list due to their characteristics: high sensitivity, high selectivity, easy miniaturization, low cost, low detection limit (in the nanomolar range), and simple operation [9, 27]. These methods also exhibit some disadvantages, including a long equilibration time and the requirement for a high pH.

Fluorescence and colorimetric probes possess numerous advantages, including low cost, visualization opportunity, noninvasive nature, high sensitivity, and biocompatibility with living systems, highlighting their important role in detecting H_2_S in biological systems [28, 29, 30]. The fluorescent methods have the best performance in real‐time H_2_S monitoring; therefore, many small‐molecule fluorescent probes have been developed for real‐time detection of H_2_S in physiological conditions.

The general structure of a potential fluorescent probe consists of three main structural motifs: fluorophore, recognition group or chemical reactive site for the target analyte, and, optionally, a linker. Potential fluorescent probes must possess some characteristics, including chemical stability, photostability, and ease of structural modifications. In the literature, the six most common fluorophores are listed, namely, rhodamine, cyanine, and hemicyanine, boron‐dipyrromethene (BODIPY), aggregation‐induced emission fluorogens, coumarin dyes, and 1,8‐naphthalimides (1,8‐NI) [28, 31]. Moreover, 1,8‐NI are among the most used fluorophores in many fluorescent probes. They are a class of fluorophores widely used in different fields, including analytical chemistry, materials, and biochemistry. They are widely represented as the basic structure of the fluorescent probe because of their characteristics like high photostability, easy preparation and structural modification, high fluorescence quantum yield, and large Stokes shift [32]. The 1,8‐NI poses electron deficiency where the imide part has an electron acceptor role, whereas the naphthalene ring has the role of п‐bridge substituents of a different type [31, 33]. The optical properties can be modulated by introducing different substituents on the naphthalene ring. Introducing electron‐donating groups like amino or hydroxyl groups at position C‐4 results in a highly fluorescent structure due to an intramolecular charge transfer (ICT), whereas electron‐withdrawing groups like nitro or azido decrease the fluorescence efficiency and result in weak fluorescence [34]. Bearing that in mind, the 1,8‐NI scaffold was a starting point in the development process of our target fluorescent probe.

The donor–acceptor interaction in the 1,8‐NI structure between the electron‐acceptor carbonyl group of the imide structure and the electron‐donating substituent at position C‐4 enables the polarization of the 1,8‐NI molecule. This polarization produces fluorescent emissions of shades of blue, yellow‐green, and even orange‐red. Based on these facts, a very important modification of the 1,8‐NI scaffold to increase the fluorescence intensity implies the conversion of the electron acceptor into an electron‐donating group [32]. For that purpose, many recognition groups and chemical reactive sites are developed, and their structure depends on the type of target analyte. According to the literature, for H_2_S detection, azide to amine approach, nucleophilic addition approach, copper displacement approach, and nitro to amine reduction approach are most commonly used [35]. As one of the selective reactive sites for H_2_S detection is the azide group, it has been widely represented in the fluorescent probe structure. The azide group can mask the fluorescence in the potential fluorophore due to its electron‐withdrawing properties. In the case of the rhodamine scaffold, reducing azide into amine in the presence of H_2_S enables the conjugation of the aromatic rings, resulting in a highly fluorescent probe [36]. The most important advantages of azide‐based probes include high selectivity for H_2_S compared with other biologically important biothiols such as cysteine, homocysteine, and glutathione [37]. Guided by that knowledge, introducing the azide group to position C‐4 of the 1,8‐NI scaffold as a chemically reactive site for target analyte H_2_S was applied in the synthetic approach of the fluorescent probe targeted in our research.

The fluorescent probes are based on selective molecular interactions, which cause changes in the chemical and optical properties of the probe used to detect the target analyte. Large amounts of molecular interaction–based probes are developed, with different mechanisms of action such as photoinduced electron transfer (PET) [38], ICT [39, 40] and fluorescence resonance energy transfer (FRET) [29]. The literature described the advantages of ICT‐based fluorescent probes, such as the plausibility of ratiometric detection and the ability to eliminate environmental and instrument distractions. In this research, our group's approach implied the usage of a simple ICT‐based fluorescent probe for H_2_S detection in human blood serum. This type of fluorescent probe development implied the binding of the azide group as the reactive site for H_2_S detection directly on the 1,8‐NI system without using a linker. A PET‐based fluorescent probe consists of fluorophore, recognition group or chemical reactive site, and short spacer, which connects these structural motifs, whereas a FRET‐based probe consists of two fluorophores, an energy donor and an energy receptor connected with unconjugated chemical bonds [41].

**FIGURE 1:**
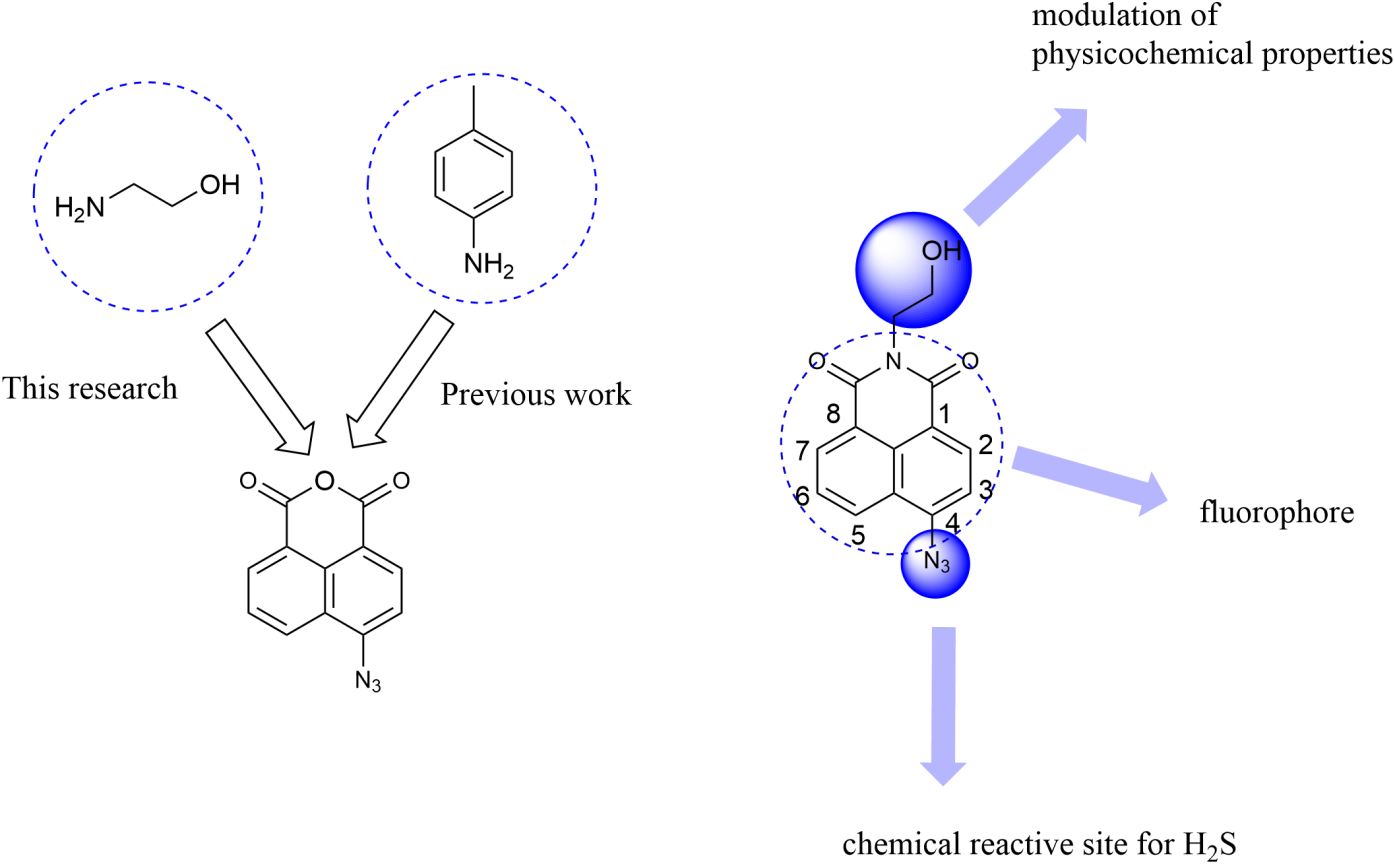
The synthetic approach of target fluorescent probe.

One of the objectives of this research implied the usage of the 1,8‐NI‐based fluorescent probe with improved water solubility (log *p* = 1.88) [42] in comparison with the fluorescent probe prepared in our previous work [43] (log *p* = 3.37) [42] to detect H_2_S in human blood serum (Figure 1). Using this fluorescent probe design is possible to investigate the influence of improved solubility on the detection performance of the prepared probe. Water solubility is a key feature of chemical compounds for its application in biological systems [44]. The fluorophore 1,8‐NI structural motif was selected, whereas the azide group was selected as a chemical reactive site for the target analyte, H_2_S. The satisfactory results were obtained with a previously published 1,8‐NI fluorescent probe with azide group as reactive site for H_2_S and *p*‐toluidine moiety on the imide part of the molecule [43]; our group's approach was the preparation of a structurally modified fluorescent probe. This structural modification implied the introduction of ethanolamine moiety on the imide part of the molecule. Based on the literature review, it was found that Yan et al. [45] and Xu et al. [46] prepared structurally the same compound, 1,8‐NI with ethanolamine moiety on the imide part of the molecule and azide group as chemical reactive site for H_2_S to apply it in bioimaging. In addition, to the best of our knowledge, this fluorescent probe has not previously been used to detect H_2_S in human serum. The UV‐Vis spectrophotometry method using methylene blue was applied for H_2_S determination on the same human serum sample as a reference method to demonstrate the applicability and accuracy of the developed fluorescent method.

## 2 Experimental

### 2.1 Materials and Methods

Reagents and solvents for the synthesis of compounds were purchased from commercial sources. Thin‐layer chromatography (TLC, solvents, and proportions are given in the text) was performed on Fluka silica gel (60 F254) plates (0.25 mm), Steinheim, Germany. For all experiments, different solvents were used, namely, dimethylformamide (DMF), ethyl acetate (EtOAc), hexane (Hx), ethanol (EtOH), and acetonitrile (ACN).

Visualization was achieved using UV light at 254 nm. Melting points were determined using Stuart Digital Melting Point Apparatus SMP 20, in open capillaries and are uncorrected. ^1^H and ^13^C NMR spectra were recorded at room temperature on Bruker Avance III HD spectrometer at 500 and 125 MHz using DMSO‐d_6_. Chemical shifts (*δ*) are given in parts per million (ppm) downfield from tetramethylsilane as an internal standard (s = singlet, d = doublet, t = triplet, and dd = doublet of doublets) (Figures –S7). C, H, and N analyses were performed on a Fisons EA 1110 CHNS elemental analyzer (Fisons Instruments, Milan, Italy) (Table S1). The excitation and emission spectra were recorded by Hitachi F‐4500 fluorescence spectrophotometer (Hitachi, Tokyo, Japan [*λ*ex = 430 nm, *λ*em = 520 nm, slit widths: 5 nm/10 nm]), whereas other fluorescence measurements were performed by Avantes Sensline AvaSpec‐ULS‐TEC. Vacutainer tubes containing a clot activator (BD Vacutainer, Becton, Dickinson, and Company, Franklin Lakes, NJ, USA) and centrifuge (Rotina 380, Hettich GmbH & Co. KG, Tuttlingen, Germany) were used in the human serum isolation procedure.

### 2.2 Synthesis

#### 2.2.1 Preparation of 6‐Azido‐1H,3H‐benzo[de]isochromene‐1,3‐dione (**1**)

4‐Bromo‐1,8‐naphthalic anhydride (150 mg, 0.54 mmol) was dissolved in dry DMF (2 mL), and the reaction mixture was stirred at room temperature for 10 min. NaN_3_ (105 mg, 1.62 mmol) was added to the reaction mixture and stirred for 2 h at room temperature. Reaction monitoring was achieved by TLC (EtOAc/Hx, 1:1). After the completion of the reaction, the reaction mixture was poured into ice, and the precipitate was filtered off and then washed with water. Compound **1** (106 mg, 82%) was obtained as a yellow solid. m.p. = 187.5°C–188°C, *R*_f_ = 0.69 (EtOAc/Hx, 1:1).

^1^H NMR (500 MHz, DMSO‐d_6_) *δ*/ppm: 7.80 (d, 1H, *J* = 8.0 Hz, H‐3), 7.92 (t, 1H, *J* = 7.8 Hz, H‐6), 8.52 (dd, 2H, *J* = 3.3, 8.2 Hz, H‐5, H‐7), 8.57 (d, 1H, *J* = 7.1 Hz, H‐2).

^13^C NMR (125 MHz, DMSO‐d_6_) *δ*/ppm: 115.244, 116.841, 119.712, 124.117, 128.120, 129.994, 131.274, 133.780, 133.809, 144.644, 160.577, 161.160. Anal. calc. for C_12_H_5_N_3_O_3_ C, 60.26; H, 2.11; N, 17.57; O, 20.07%; Found: C, 58.90; H, 3.40; N, 16.48; O, 21.22%.

#### 2.2.2 Preparation of 6‐Azido‐2‐(2‐hydroxyethyl)‐1*H*‐benzo[*de*]isoquinoline‐1,3(2*H*)‐dione (**2**)

Compound **1** (100 mg, 0.41 mmol) was dissolved in EtOH (10 mL) under reflux. After dissolving compound **1**, ethanolamine (25 mg, 24.8 μL, 0.41 mmol) was added, and the reaction mixture was refluxed for 6 h. The reaction was monitored by TLC (EtOAc/Hx, 1:1). The reaction mixture was cooled to room temperature, and the crude product precipitated, filtered off from the reaction mixture. Corresponding 1,8‐NI derivative, compound **2** (63.8 mg, 56%), brown solid was obtained. m.p. = 169.1°C–170.1°C, *R*_f_ = 0.41 (EtOAc/Hx, 1:1).

^1^H NMR (500 MHz, DMSO) *δ*/ppm: 3.65 (q, 2H, *J* = 5.9 Hz, 2‐Et), 4.14 (t, 2H, *J* = 6.6 Hz, 1‐Et), 4.82 (t, 1H, *J* = 5.4 Hz, OH), 7.70 (d, 1H, *J* = 8.0 Hz, H‐3), 7.83 (t, 1H, *J* = 8.0 Hz, H‐6), 8.37 (d, 1H, *J* = 8.3 Hz, H‐7), 8.42 (d, 1H, *J* = 8.0 Hz, H‐5), 8.49 (d, 1H, *J* = 6.9 Hz, H‐2).

^13^C NMR (125 MHz, DMSO) *δ*/ppm: 42.3, 58.28, 116.36, 118.75, 122.72, 123.96, 127.72, 128.70, 131.87, 131.97, 143.17, 163.33, 163.79.

Anal. calc. for C_14_H_10_N_4_O_3_ C, 59.57; H, 3.57; N, 19.85; O, 17.00%; Found: C, 59.25; H, 4.39; N, 17.39; O, 18.97%.

### 2.3 General Procedure for Fluorescence Spectra Measurements

Absolute EtOH was used for the preparation of solutions of compound **2** (*c* = 10 μmol L^−1^), whereas for the preparation of solutions of Na_2_S, KH_2_PO_4_, K_2_HPO_4_, Na_2_SO_4_, Na_2_SO_3_, KSCN, GSH, and cysteine ultrapure water was used. For the fluorescent characterization of compound **2** and its reduced form, their EtOH stock solution was used; excitation spectra (λem. = 520 nm) and emission spectra (λexc. = 430 nm) are presented in Figure S8. Spectra were recorded with the corresponding spectrophotometer with slit widths of exc. 5 nm/emm. 10 nm. Before the measurement, all samples were incubated at 25°C for 15 min. Ultimately, Na_2_S was used as a source of H_2_S. All experiments were carried out three times.

### 2.4 Application of Compound **2** for Detection of H_2_S in Human Blood Serum

For the purpose of human serum isolation, venous blood samples were drawn into 6‐mL vacutainer tubes containing a clot activator. After collection, the samples were centrifuged at 3600 RPM for 10 min at 4°C to separate the serum. The resulting serum was then divided into the required number of aliquots, which were transferred to microcentrifuge tubes. These aliquots were carefully stored at −80°C. Serum collection and isolation were conducted at the Department of Physiology and Immunology (Laboratory for Molecular and Clinical Immunology), Faculty of Medicine, Osijek, Croatia.

To detect H_2_S in human serum using a fluorescent probe, ACN (1 mL) was added to the collected aliquot (500 μL) to precipitate the protein in serum [47]. After the precipitation of proteins, a clear supernatant was diluted to 5 mL using distilled water. A clear supernatant (250 μL), PBS buffer (250 μL, pH = 7.4), Na_2_S (250 μL, 5, 10, 15, and 20 μmol L^−1^), and a solution of compound **2** (250 μL, 10 μmol L^−1^) were added to four Eppendorf tubes. For recording the 0 point (blank), a clear supernatant (250 μL), PBS buffer (250 μL, pH = 7.4), ZnCl_2_ (250 μL, 100 μmol L^−1^), and a solution of compound **2** (250 μL, 10 μmol L^−1^) were added to an Eppendorf tube. For recording the X point, instead of ZnCl_2_, distilled water (250 μL) was added to the Eppendorf tube.

All six Eppendorf tubes were incubated for 30 min at 37°C. Ultimately, before measurement, absolute EtOH (1 mL) was added to all six Eppendorf tubes.

### 2.5 Application of UV‐Vis Spectrophotometry Method Using Methylene Blue for H_2_S Detection in Human Blood Serum

Into the aliquot of human blood serum (500 μL) ACN (1 mL) was added. After the precipitation of proteins, a clear supernatant was diluted to 5 mL using distilled water. Into an Eppendorf tube, diluted supernatant (250 μL), trichloroacetic acid (300 μL, 10%), zinc acetate (150 μL, 1%), *N*,*N*‐dimethyl‐*p*‐phenylenediamine sulfate in 7.2 mol L^−1^ HCl (100 μL, 20 μmol L^−1^), and FeCl_3_ in 1.2 mol L^−1^ HCl (133 μL, 30 μmol L^−1^) were added. The absorbance of the resulting solution was measured 15 min later. All samples were spiked with the same concentrations as in the fluorescent method, although the concentration of H_2_S was calculated from a calibration curve.

## 3 Results and Discussion

### 3.1 Chemistry

**SCHEME 1:**
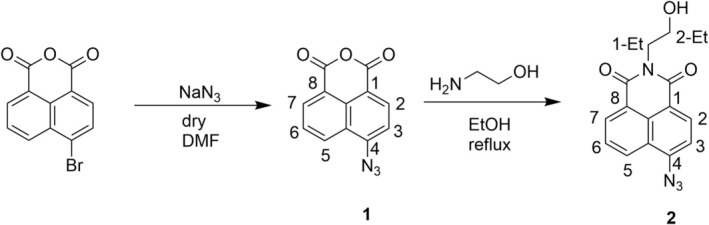
Synthetic route for the preparation of compound **2** and NMR numbering scheme.

This work implied the development and synthesis of the 1,8‐NI derivative, fluorescent probe, compound **2**. The synthesis of compound **2** was carried out according to Zhang et al. [48]. The literature‐described procedure implied two reaction steps, whereby, in the case of this work, the first reaction step was modified. This modification included the addition of 3 equiv. of NaN_3_ and the addition of NaN_3_ to the solution of 4‐bromo‐1,8‐naphthalic anhydride without previous dissolving in water. The synthetic route carried out for the preparation of the target compound **2** is described in Scheme 1. As a starting compound, the commercially available 4‐bromo‐1,8‐naphthalic anhydride was used. The first reaction step implied nucleophilic aromatic substitution on the starting compound with the aim of introducing the azide group as a chemically reactive site for H_2_S in the 1,8‐NI structure, whereby compound **1** was synthesized with a satisfactory yield of 82%. The second reaction step included the binding of the ethanolamine moiety on the *N*‐position of the 1,8‐NI structure, which resulted in the formation of the target compound **2**, with a yield of 56%. The synthesized compounds **1** and **2** were characterized by ^1^H and ^13^C NMR spectroscopy and elemental analysis (Figures –S7 and Table S1).

### 3.2 Investigation of Effect of pH and Time Dependence of Compound **2**

**FIGURE 2:**
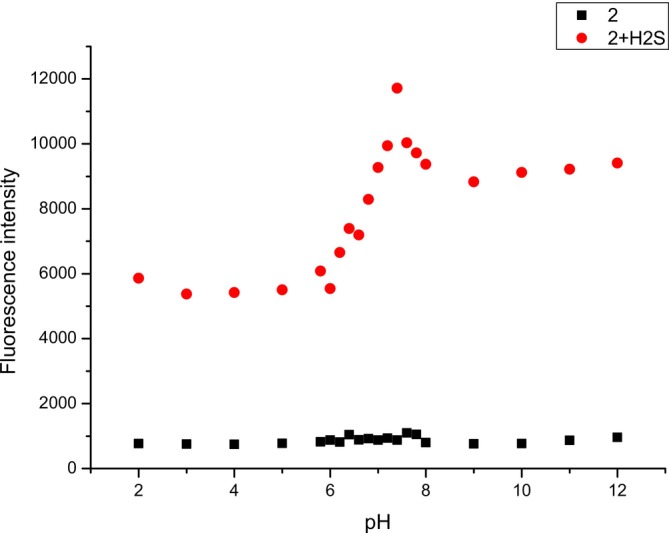
Fluorescence intensities of compound **2** (10 μmol L^−1^) at 520 nm with and without addition of H_2_S at pH range of 2–12.

For the purpose of investigating the detection performance of compound **2**, the influence of pH change on fluorescence intensity was studied (Figure 2 and Table S2). An experiment was carried out in PBS buffer in the pH range 2–12, with a large number of measurements in the range of 5.8–8, slightly above and below the biologically relevant pH range, as the pH of human serum is 7.35–7.45 [49]. Compound **2**, without the addition of H_2_S, was not sensitive to the biologically relevant pH range over 2–12. After the addition of H_2_S, an enhanced fluorescence peak at 520 nm appeared in the range of 6.8–7.6, whereas the maximum peak of fluorescence intensity appeared at a pH of 7.4. Considering the literature value of the pH of human serum, it can be concluded that compound **2** has great potential to be used for H_2_S detection in the pH conditions of human serum (Figure 2).

**FIGURE 3:**
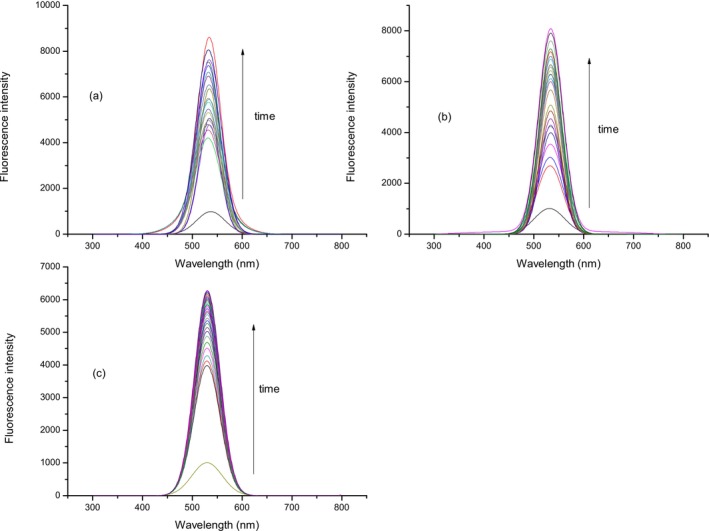
Fluorescence intensity of compound **2** (10 μmol L^−1^) after treatment with H_2_S (5 μmol L^−1^). Fluorescence intensity was recorded at 520 nm. Time points represent 0–60 min; after H_2_S addition, spectra were recorded every 2 min. Experiment was conducted at (a) pH = 7.0, (b) pH = 7.2, and (c) pH = 7.4.

**FIGURE 4:**
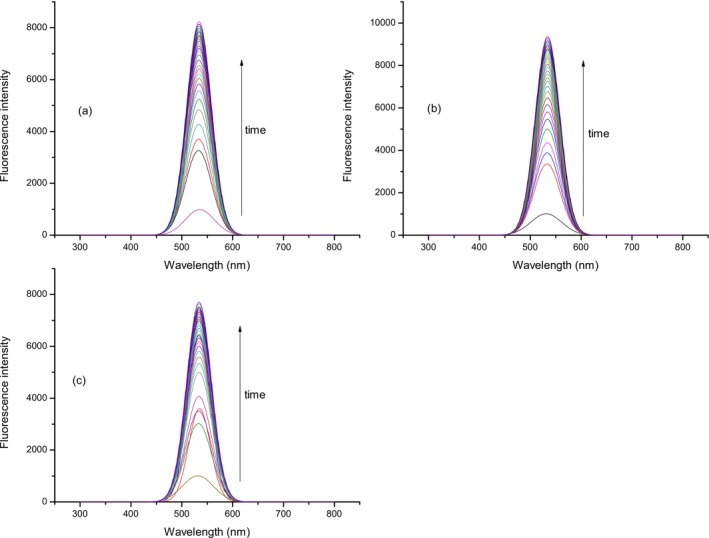
Fluorescence intensity of compound **2** (10 μmol L^−1^) after treatment with H_2_S (10 μmol L^−1^). Fluorescence intensity was recorded at 520 nm. Time points represent 0–60 min; after H_2_S addition, spectra were recorded every 2 min. Experiment was conducted at (a) pH = 7.0, (b) pH = 7.2, and (c) pH = 7.4.

**FIGURE 5:**
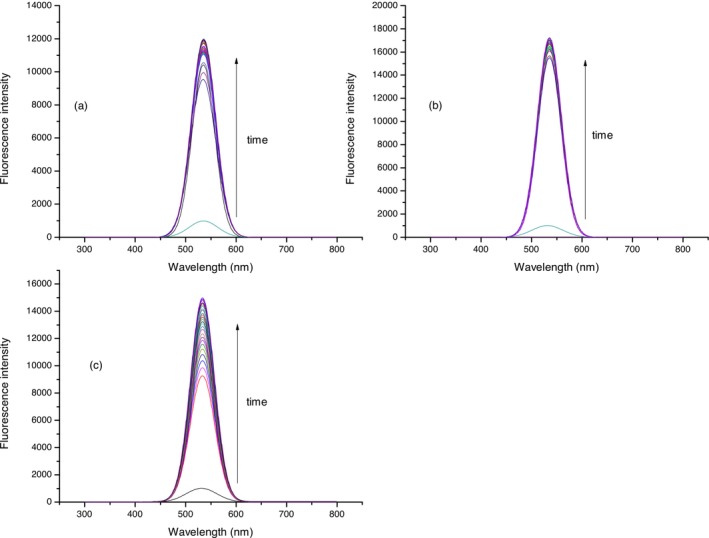
Fluorescence intensity of compound **2** (10 μmol L^−1^) after treatment with H_2_S (20 μmol L^−1^). Fluorescence intensity was recorded at 520 nm. Time points represent 0–60 min; after H_2_S addition, spectra were recorded every 2 min. Experiment was conducted at (a) pH = 7.0, (b) pH = 7.2, and (c) pH = 7.4.

Solution of compound **2** (10 μmol L^−1^) was treated with three different concentrations of H_2_S (5, 10, and 20 μmol L^−1^) during 1 h, 0–60 min after H_2_S addition with the aim of investigating the fluorescent response of compound **2** to H_2_S. Spectra were recorded every 2 min (Figures 3, 4, 5) at three biologically relevant pH values (7–7.4) for all three concentrations of H_2_S, considering that the percentage composition of sulfide in plasma is sensitive to the pH [50]. During the period of 1 h in the case of all three chosen concentrations of H_2_S at selected pH values, an increase in fluorescence intensity was observed.

**FIGURE 6:**
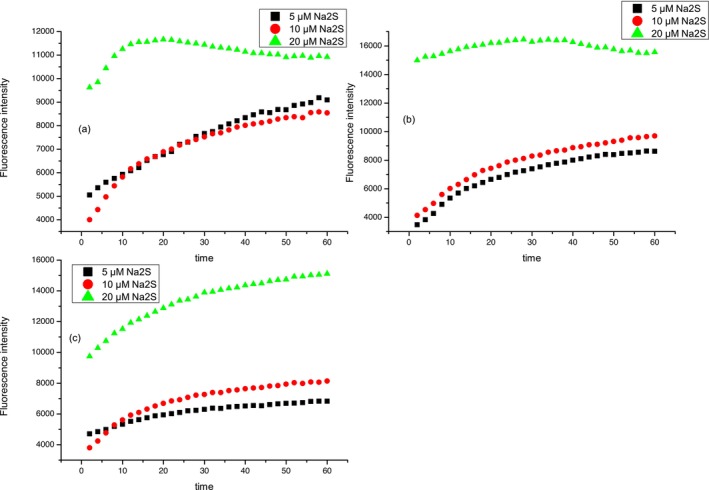
Fluorescence intensity of compound **2** (10 μmol L^−1^) after treatment with H_2_S (10 and 20 μmol L^−1^). Fluorescence intensity was recorded at 520 nm. Time points represents 0–60 min; after H_2_S addition, spectra were recorded every 2 min. Experiment was conducted at (a) pH = 7.0, (b) pH = 7.2, and (c) pH = 7.4.

Fluorescence intensities of compound **2** (10 μmol L^−1^) at 520 nm after treatment with three different concentrations of Na_2_S (5, 10, and 20 μmol L^−1^) at three pH values (7–7.4) in the reaction period of 1 h are investigated. In the case of all three pH values (a–c), the same trend is observed. The fluorescence intensity of probe **2** after treatment with all three selected concentrations of Na_2_S increased during the time and became constant after approximately 30–40 min (Figure 6). From Figure 6, it is evident that in the case of 20 μmol L^−1^, Na_2_S fluorescence intensity was higher and increased during the period of approximately 50 min for all three pH values. This trend can be explained by mechanistic insight for reducing aryl azides with H_2_S. Henthorn and Pluth [51] explained that HS^−^ is the reactive species required for the aryl azide reduction whereby two HS equivalents are required for complete reduction of the azide group.

### 3.3 Investigation of Selective Response of Compound **2** to H_2_S

**FIGURE 7:**
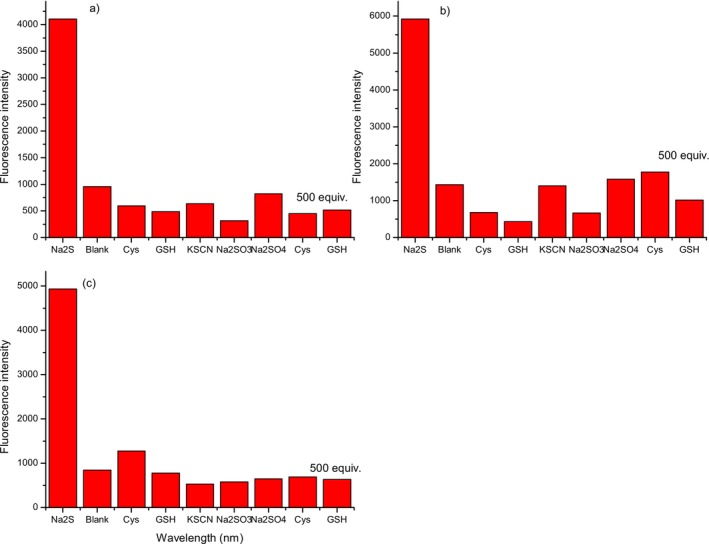
Fluorescence intensity response of compound **2** to selected analytes at three biological relevant pH values of (a) pH = 7.0, (b) pH = 7.2, and (c) pH = 7.4.

**FIGURE 8:**
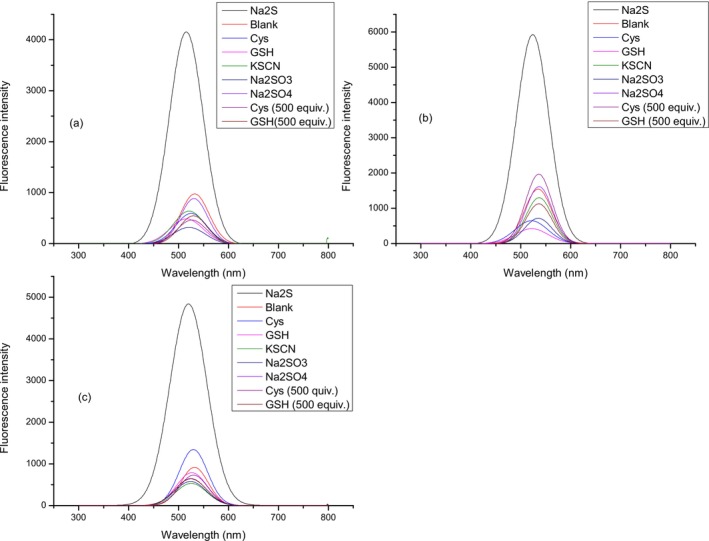
Fluorescent emission spectra of compound **2** after treatment with selected analytes at three biological relevant pH values of (a) pH = 7.0, (b) pH = 7.2, and (c) pH = 7.4.

To confirm the ability of probe **2** to detect H_2_S and to investigate the possibility of its application in biological systems, more precisely human blood serum, the selectivity of compound **2** for H_2_S detection was examined. For that purpose, as analytes (50 equiv.), except Na_2_S, KSCN, Na_2_SO_3_, and Na_2_SO_4_, biologically important thiols cysteine (Cys) and glutathione (GSH) were chosen (Figures 7 and 8 and Table S3). Moreover, with the aim of additionally confirming the ability of probe **2** to selectively detect H_2_S in human serum, experiments were also carried out in the presence of 500 equiv. Cys and GSH. From Figures 7 and 8, it is evident that the change in fluorescence intensity after treatment with other analytes was insignificant in comparison with Na_2_S. These results indicate the possibility of the application of probe **2** for selective H_2_S detection in human blood serum.

### 3.4 Investigation of Influence of Concentration of Na_2_S on Fluorescence Intensity

**FIGURE 9:**
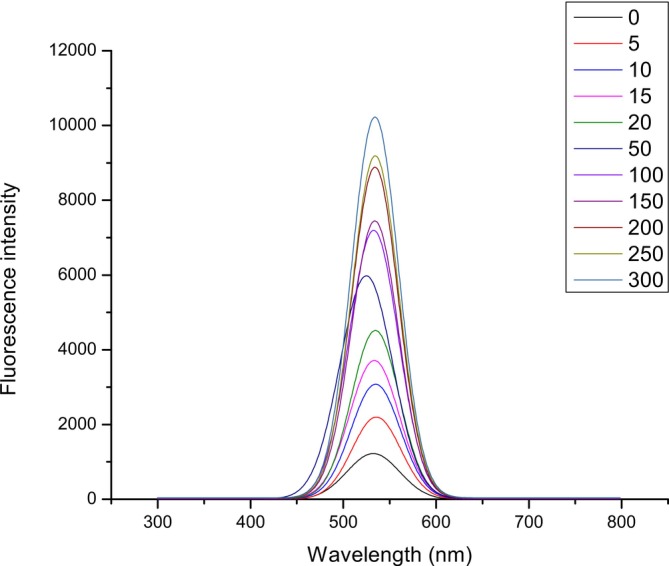
Fluorescent emission spectra of compound **2** after treatment with Na_2_S in the concentration range of 0–300 μmol L^−1^.

The reduction of compound **2** (10 μmol L^−1^) was carried out with different concentrations of H_2_S in the range of 0–300 μmol L^−1^ for the purpose of examining the detection performance of the fluorescent probe, mainly, detection limit calculation. The mentioned range is selected according to the literature‐described concentration range of H_2_S in blood or plasma 10–300 μmol L^−1^ [52]. Fluorescent emission spectra of compound **2** after treatment with the mentioned concentration of Na_2_S are recorded and presented in Figure 9.

The dependence of fluorescence intensity on mentioned concentrations was examined, and the corresponding graph was plotted (Figure S9). From the plotted graph, it was observed that in the concentration range of 0–20 μmol L^−1^, an excellent linearity relationship was obtained although increasing the concentration caused a significant lack of linearity. One of the possible explanations for that trend can also imply the mechanistic insight [51] according to which for complete reduction of compound **2** with H_2_S, 2 equiv. of H_2_S are required.

For the prepared probe, compound **2**, the detection limit (3σ/k) was calculated after 30 min incubation with H_2_S in PBS buffer at pH = 7.4. The value of the detection limit was found to be 0.16 μmol L^−1^ (Figure S9). According to the literature evidence for this fluorescent probe, prepared by Yan et al., a minimum concentration was calculated to be 1 × 10^−7^ mol·L^−1^, whereas in the case of research by Xu et al. [46], the detection limit was calculated to be 1.09 μmol L^−1^. These results can be compared to our obtained value of the detection limit. Furthermore, for the structurally similar compounds, the detection limit was found to be in the range of 0.02–0.7 μmol L^−1^ [48, 53, 54]. More precisely, in the case of the 1,8‐NI‐based probe with metoxyaniline moiety on the imide part of the molecule, the detection limit was calculated to be 0.017 μmol L^−1^ [53]. In addition, in the instance of the hydroxyaniline‐functionalized probe, the detection limit was calculated to be 0.051 μmol L^−1^ [48]. In both cases, the values of the detection limit are lower in comparison with compound **2** from this research. But, in the case of the propyl group functionalized 1,8‐NI probe [54], the calculated value of the detection limit was found to be 0.74 μmol L^−1^, which is higher in comparison with compound **2** from this research. On the basis of this comparison, and considering the research from Yan et al. [45] and Xu et al. [46] for the structurally the same probe, it can be concluded that the results from our research are comparable with those obtained in the literature‐described research.

The detection limit of the previously prepared probe by our group was found to be 0.085 μmol L^−1^. Because in the case of this probe development we were guided by the idea of the decreasing log *P*, in order to increase solubility and ultimately to decrease the detection limit, we compared the obtained results with those from previous work [43]. On the basis of the obtained results, it can be concluded that the increase in water solubility does not have a significant influence on the detection performance of the probe for the purpose of H_2_S detection in human blood serum.

### 3.5 Application of Compound 2 in Quantitative Detection of H_2_S in Human Serum and Comparison With UV‐Vis Spectrophotometry Method Using Methylene Blue

The prepared fluorescent probe **2** was successfully applied in H_2_S detection in human blood serum. The standard addition method was used for confirmation of the accuracy of the H_2_S determination, whereby Na_2_S was spiked as an internal standard. The selected concentrations of Na_2_S used for spiking human blood serum samples were chosen to be 5, 10, 15, and 20 μmol L^−1^ due to the linear relationship between fluorescence intensity and concentration in the concentration range of 5–20 μmol L^−1^. For the 0 point (blank), ZnCl_2_ was added to a human serum sample in order to remove H_2_S from the sample [55]. All samples were incubated at 37°C after which fluorescence spectra were measured. The concentration of H_2_S in the human serum sample was found to be 16.2 μmol L^−1^, whereby the concentration was calculated as the ratio of intercept and slope (Figure S10). In Table 1, the spiked level of Na_2_S in the human serum sample and the recovery values are presented. Considering the literature value of physiological serum concentration of H_2_S, which is in the range of 10–300 μmol L^−1^ [52], it can be observed that there is accordance of our obtained results with those described in the literature. In comparison with the literature‐described results, the obtained concentration of H_2_S in the human serum sample in this research is lower. This fact can be explained by the transient nature of H_2_S and its low concentration in different tissues [52, 55].

**TABLE 1:** Determination of H_2_S in human serum sample using standard addition method in fluorescent probe detection and UV‐Vis spectrophotometry method using methylene blue.

		Found (μmol L ^−1^ )		Recovery (%)		RSD (%)	
	Added (μmol L^−1^)	Fluorescence	Methylene blue	Fluorescence	Methylene blue	Fluorescence	Methylene blue
Human serum	5	4.4	5.2	88	104	9	3
	10	9.8	10.1	98	101	1	1
	15	15	14.5	100	97	0	2
	20	20.2	20.3	101	102	1	1

Abbreviation: RSD, relative standard deviation.

In order to investigate the applicability and the accuracy of the developed method, UV‐Vis spectrophotometry method using methylene blue, as the most commonly used method for sulfide measurement in biological samples, was applied. This method implies the reaction of *N*,*N*‐dimethyl‐*p*‐phenylenediamine, sulfide, and FeCl_3_ in order to produce methylene blue dye, which can be detected spectrophotometrically [56, 57]. The described method was applied to the same human serum sample as in the case of the application of fluorescent probe **2**. The concentration of H_2_S was found to be 17.1 μmol L^−1^, whereby concentration was calculated as the ratio of intercept and slope (Figure S10).

## 4 Conclusion

A novel fluorescent probe for the purpose of H_2_S detection in human blood serum was prepared. The prepared fluorescent probe, compound **2**, in its structure possesses 1,8‐NI scaffold as fluorophore, azide group as chemical reactive site for H_2_S, and ethanolamine moiety for modulation of physicochemical properties, more precisely, increase of water solubility. The azide group is directly bound to a fluorophore, without the usage of a linker, based on which it can be concluded that the prepared fluorescent probe is an ICT‐based probe. Several effects on fluorescence intensity were investigated, including the effect of pH and the time dependence of compound **2**, the selective response of probe **2** to H_2_S, and the influence of the concentration of Na_2_S as a source of H_2_S. The calculated detection limit for the fluorescent probe, compound **2**, was 0.16 μmol L^−1^ for the linear range of 0–20 μmol L^−1^.

To investigate the potential application of probe **2** in biological systems, compound **2** was applied for H_2_S detection in human blood serum whereby the concentration of H_2_S was found to be 16.2 μmol L^−1^. Furthermore, in order to investigate and confirm the utilization of fluorescent probe **2** for H_2_S determination in biological samples, a commonly used method for H_2_S determination was applied on the same human serum sample. Applying the UV‐Vis spectrophotometry method using methylene blue, H_2_S concentration was found to be 17.1 μmol L^−1^. The obtained results with the novel fluorescent probe **2** are in accordance with those obtained applying the UV‐Vis spectrophotometry method using methylene blue, indicating that the developed method could be used for selective and accurate H_2_S detection in biological samples, primarily in human blood serum.

## Author Contributions

**Aleksandar Széchenyi:** writing – review and editing, funding acquisition, methodology, conceptualization, investigation. **Mirela Samardžić:** validation, supervision. **Mateja Budetić:** validation, methodology. **Ines Drenjančević:** formal analysis, supervision. **Nikolina Kolobarić:** formal analysis, investigation. **Gábor Mikle:** formal analysis. **Barna Kovács:** methodology, data curation. **Andrea Dandić:** writing – original draft, investigation, methodology, conceptualization, visualization, formal analysis, data curation. All authors have critically revised the first version and have approved the final version of the manuscript.

## Conflicts of Interest

The authors declare no conflicts of interest.

## Supporting information


**Figure S1:** bio70295‐sup‐0001‐Supplementary_Material.docx. ^1^H NMR of compound **1**.
**Figure S2:** bio70295‐sup‐0001‐Supplementary_Material.docx. ^1^H NMR of compound **1**.
**Figure S3:** bio70295‐sup‐0001‐Supplementary_Material.docx. ^13^C NMR of compound **1**.
**Figure S4:** bio70295‐sup‐0001‐Supplementary_Material.docx. ^1^H NMR of compound **2**.
**Figure S5:** bio70295‐sup‐0001‐Supplementary_Material.docx. ^1^H NMR of compound **2**.
**Figure S6:** bio70295‐sup‐0001‐Supplementary_Material.docx. ^1^H NMR of compound **2**.
**Figure S7:** bio70295‐sup‐0001‐Supplementary_Material.docx. ^13^C NMR of compound **2**.
**Table S1:**. Elemental analysis of prepared compounds.
**Table S2:** Fluorescence intensities of compound **2** (10 μmol L^−1^) at 520 nm with and without addition of H_2_S at pH values in the range of 2–12.
**Table S3:** Fluorescence intensity response of compound **2** to selected analytes at three biological relevant pH values pH = 7.0, pH = 7.2 and pH = 7.4.
**Figure S8:** (a) Emission spectra of compound **2** excitation at 430 nm, (b) excitation spectra of compound **2** emission at 520 nm, (c) emission spectra of reduced form of compound **2** excitation at 430 nm, and (d) excitation spectra of reduced form of compound **2** emission at 520 nm.
**Figure S9:** Plot of the fluorescence intensity of compound **2** (10 μmol L^−1^) in the (a) concentration range of 0–20 μmol L^−1^ of H_2_S at 520 nm and (b) concentration range of 0–300 μmol L^−1^ of H_2_S at 520 nm.
**Figure S10:** Determination of H_2_S concentration in spiked human serum sample using Na_2_S as internal standard (5–20 μmol L^−1^) by (a) fluorescent probe detection at 520 nm and (b) UV‐Vis spectrophotometry method using methylene blue in spiked human plasma sample using Na_2_S as internal standard (5–20 μmol L^−1^) at 570 nm.

## Data Availability

The data that support the findings of this study are available on request from the corresponding author. The data are not publicly available due to privacy or ethical restrictions.
