# Supplementary material for: Development and Application of a 1,8‐Naphthalimide‐Based Fluorescent Probe for Sensitive Detection of Hydrogen Sulfide in Human Blood Serum
Source: Luminescence. 2025 Aug 19;40(8):e70295. doi: 10.1002/bio.70295 (PMC12364621; doi:10.1002/bio.70295)
Supplement: Supplementary file 1 — Figure S1: bio70295‐sup‐0001‐Supplementary_Material.docx. 1H NMR of compound 1. Figure S2: bio70295‐sup‐0001‐Supplementary_Material.docx. 1H NMR of compound 1. Figure S3: bio70295‐sup‐0001‐Supplementary_Material.docx. 13C NMR of compound 1. Figure S4: bio70295‐sup‐0001‐Supplementary_Material.docx. 1H NMR of compound 2. Figure S5: bio70295‐sup‐0001‐Supplementary_Material.docx. 1H NMR of compound 2. Figure S6: bio70295‐sup‐0001‐Supplementary_Material.docx. 1H NMR of compound 2. Figure S7: bio70295‐sup‐0001‐Supplementary_Material.docx. 13C NMR of compound 2. Table S1:. Elemental analysis of prepared compounds. Table S2: Fluorescence intensities of compound 2 (10 μmol L−1) at 520 nm with and without addition of H2S at pH values in the range of 2–12. Table S3: Fluorescence intensity response of compound 2 to selected analytes at three biological relevant pH values pH = 7.0, pH = 7.2 and pH = 7.4. Figure S8: (a) Emission spectra of compound 2 excitation at 430 nm, (b) excitation spectra of compound 2 emission at 520 nm, (c) emission spectra of reduced form of compound 2 excitation at 430 nm, and (d) excitation spectra of reduced form of compound 2 emission at 520 nm. Figure S9: Plot of the fluorescence intensity of compound 2 (10 μmol L−1) in the (a) concentration range of 0–20 μmol L−1 of H2S at 520 nm and (b) concentration range of 0–300 μmol L−1 of H2S at 520 nm. Figure S10: Determination of H2S concentration in spiked human serum sample using Na2S as internal standard (5–20 μmol L−1) by (a) fluorescent probe detection at 520 nm and (b) UV‐Vis spectrophotometry method using methylene blue in spiked human plasma sample using Na2S as internal standard (5–20 μmol L−1) at 570 nm. [file BIO-40-e70295-s001.docx]

**Development and application of a 1,8-naphthalimide-based fluorescent probe for sensitive detection of hydrogen sulfide in human blood serum**

Aleksandar Széchenyi^1,4,5,^, Mirela Samardžić^1^, Mateja Budetić^1^, Ines Drenjančević^2^, Nikolina Kolobarić^2^, Gábor Mikle^3,4,6^, Barna Kovács^4,5^, Andrea Dandić^1^

^1^Department of Chemistry, Josip Juraj Strossmayer University of Osijek, Cara Hadrijana 8/A, 31000 Osijek, Croatia

^2^Department of Physiology and Immunology, Faculty of Medicine Osijek, J. J. Strossmayer University of Osijek, J. Huttlera 4, 31000 Osijek, Croatia

^3^Department of General and Inorganic Chemistry, Faculty of Sciences, University of Pécs, Ifjúság útja 6, 7624 Pécs, Hungary

^4^Green Chemistry Research Group, János Szentágothai Research Centre, University of Pécs, Ifjúság útja 20, 7624 Pécs, Hungary

^5^Institute of Pharmaceutical Technology and Biopharmacy, Faculty of Pharmacy, University of Pécs, Rokus utca 4, 7624 Pécs, Hungary

^6^Research Group for Selective Chemical Syntheses, HUNREN-PTE, Ifjúság útja 6., H-7624 Pécs, Hungary

**Corresponding author**

andreajuric@kemija.unios.hr (Andrea Dandić)


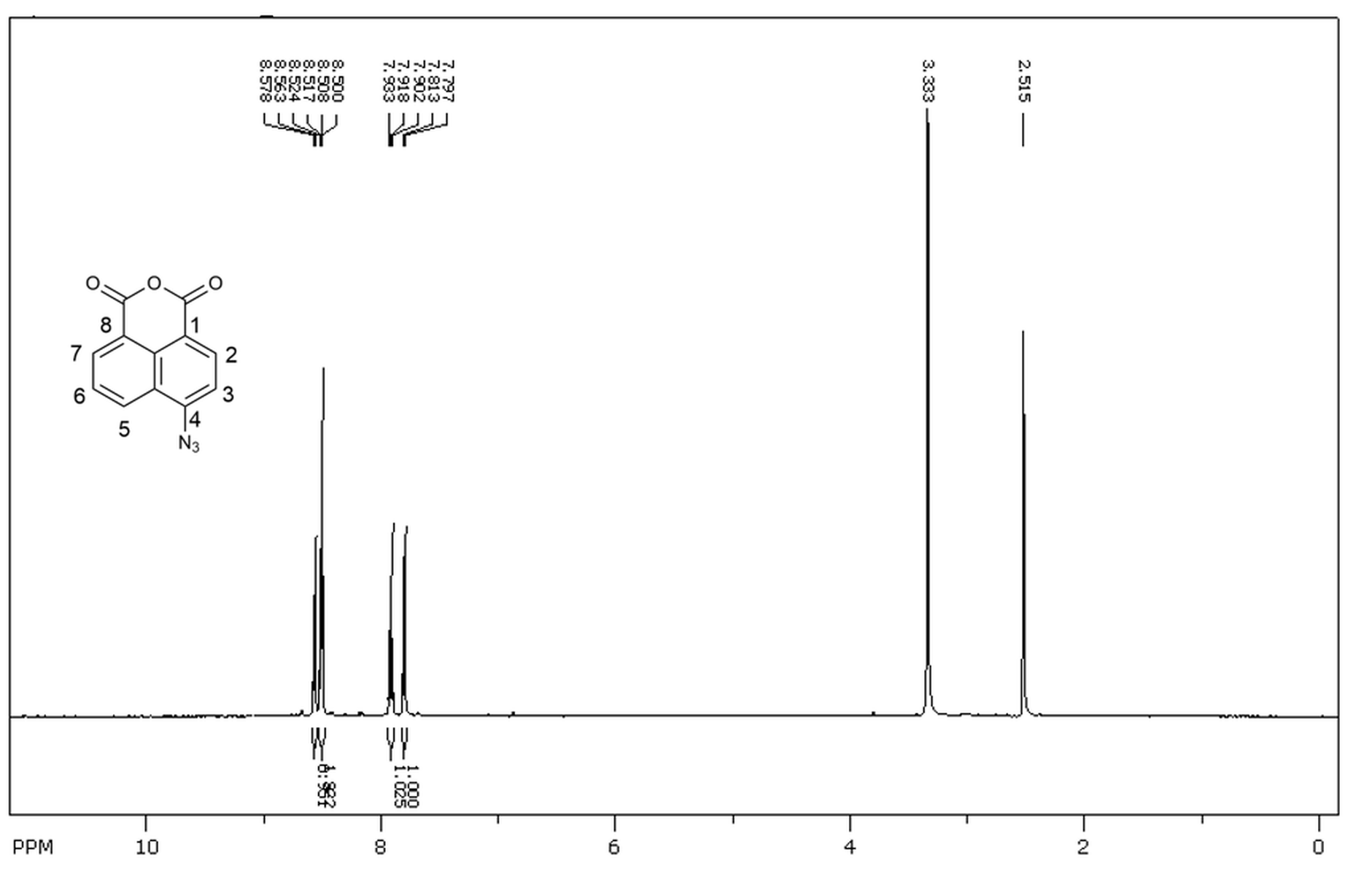


**Figure S1 |** ^1^H NMR of compound **1**.


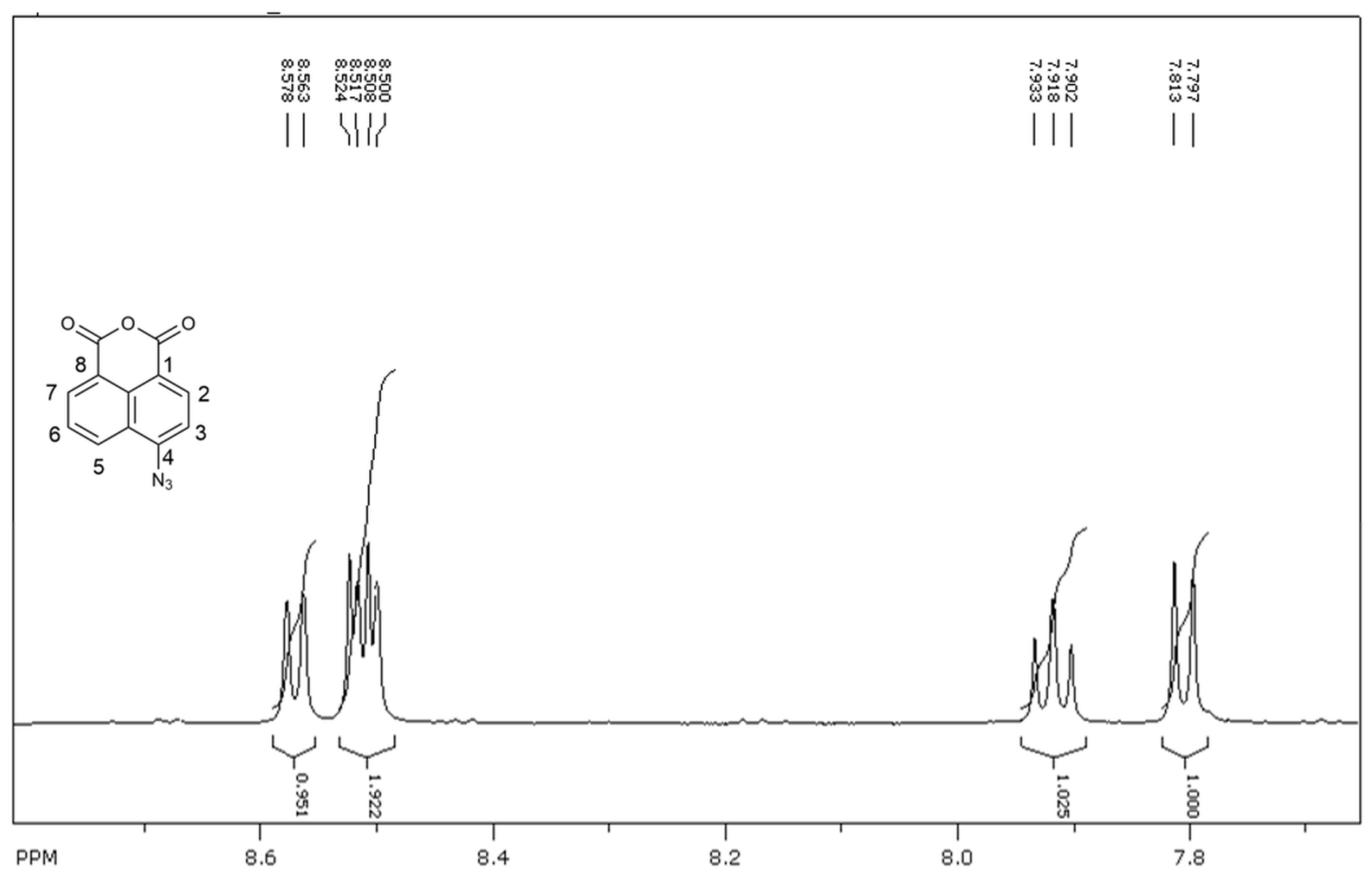


**Figure S2 |** ^1^H NMR of compound **1**.


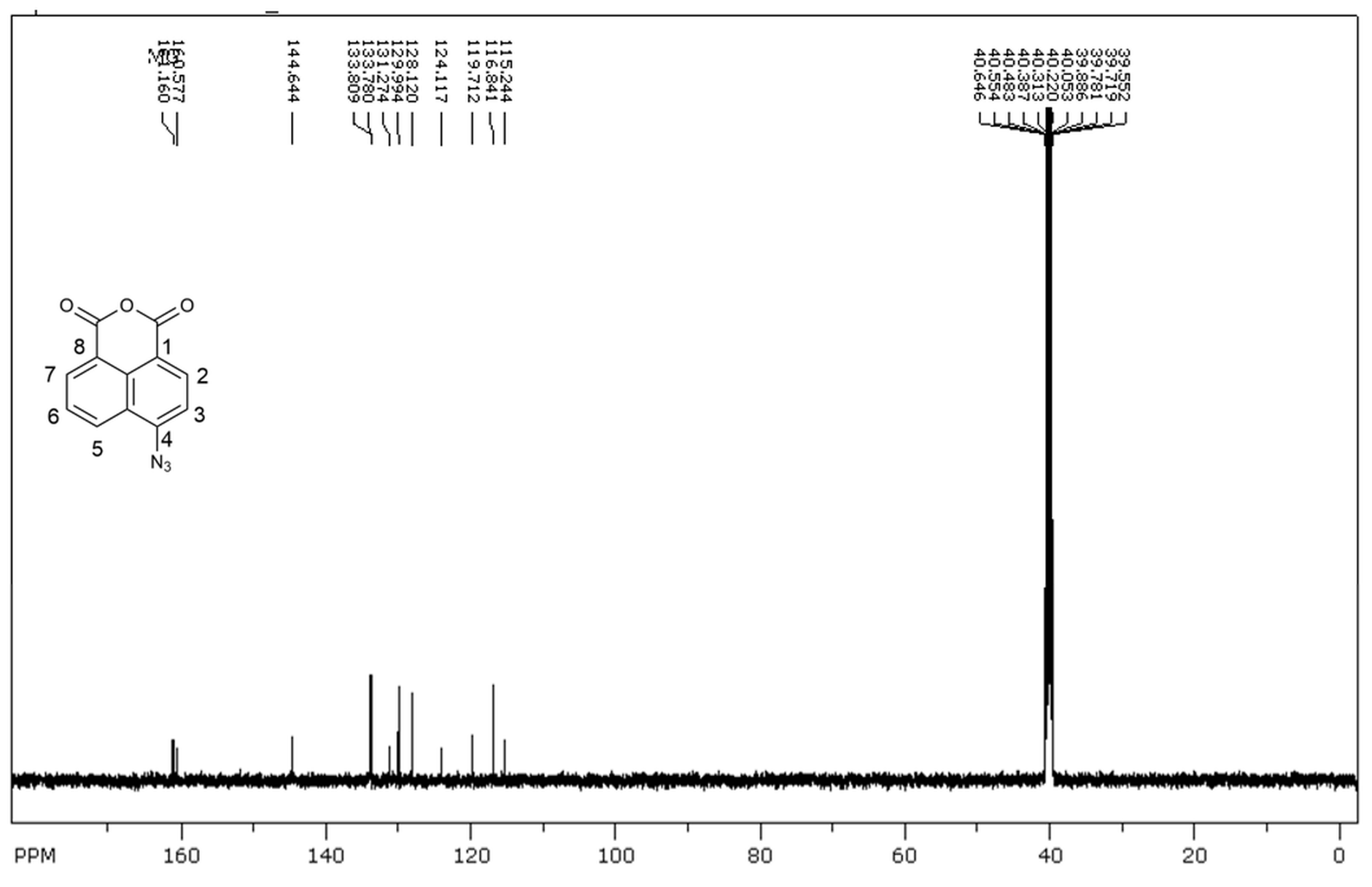


**Figure S3 |** ^13^C NMR of compound **1**.

**
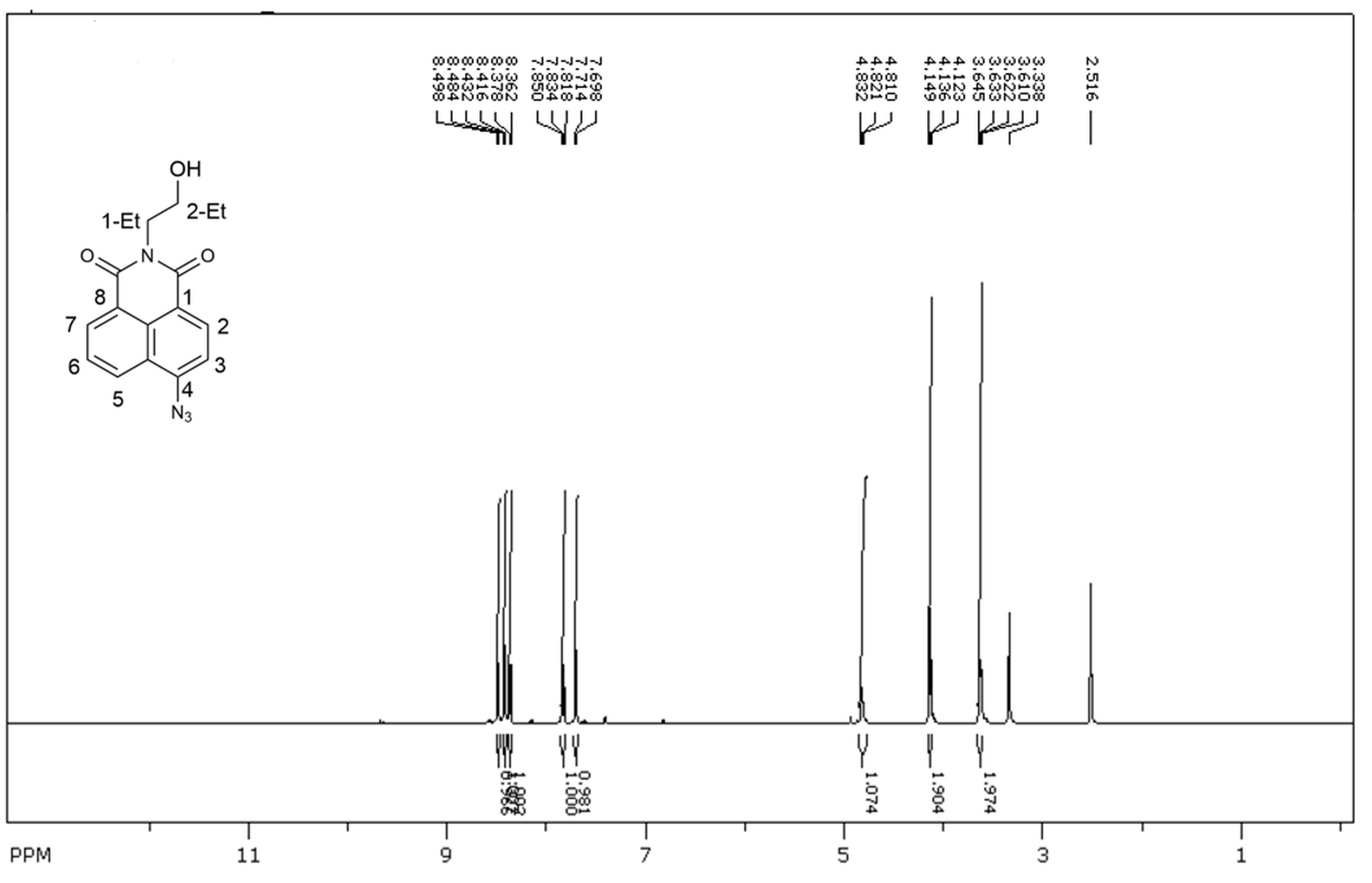
**

**Figure S4 |** ^1^H NMR of compound **2**.


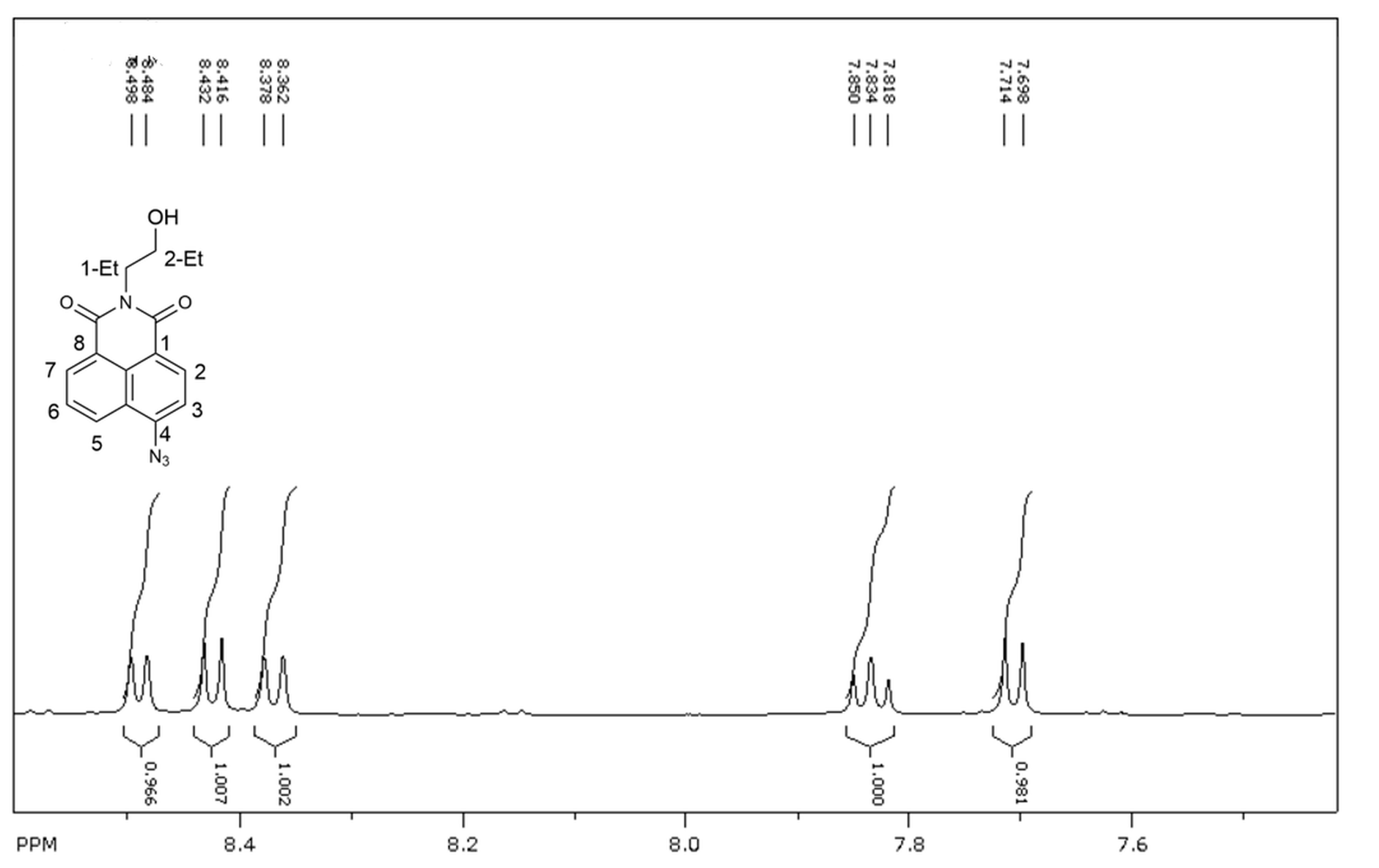


**Figure S5 |** ^1^H NMR of compound **2**.


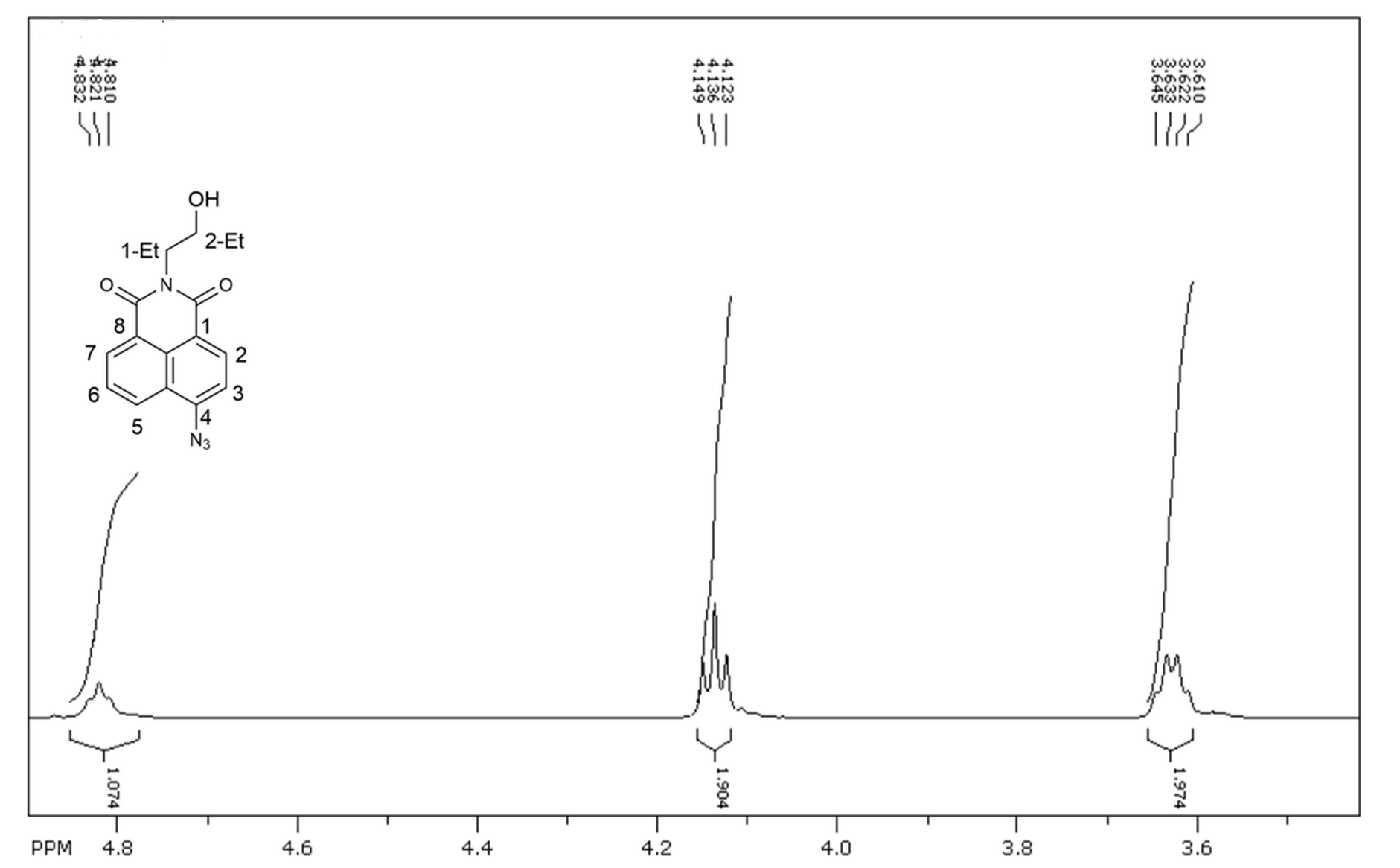


**Figure S6 |** ^1^H NMR of compound **2**.


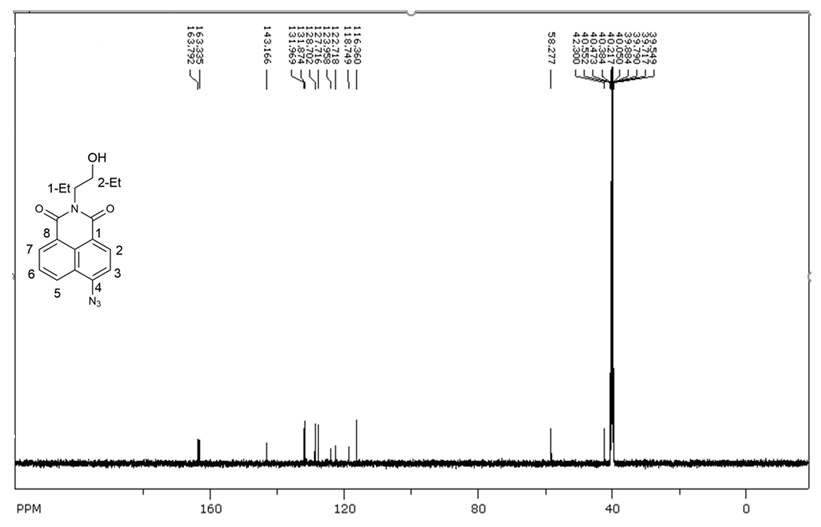


**Figure S7 |** ^13^C NMR of compound **2**.

**Table S1**

Elemental analysis of prepared compounds.

| Compound |  |
| --- | --- |
| 1 | Nitrogen 16.48 Carbon 58.90 Hydrogen 3.40 |
| 2 | Nitrogen 17.39 Carbon 59.25 Hydrogen 4.39 |

Table S2. Fluorescence intensities of compound **2** (10 μmol L^−1^) at 520 nm with and without addition of H_2_S at pH values in the range of 2–12.

| pH | without H_2_S addition | | with H_2_S addition | |
| --- | --- | --- | --- | --- |
|  | Fluorescence intensity^a^ | SD^b^ | Fluorescence intensity^a^ | SD^b^ |
| 2.0 | 749.1667 | 11.34087 | 5550.4 | 220.1674 |
| 3.0 | 755.5667 | 10.50532 | 5557.9 | 210.1557 |
| 4.0 | 755.5667 | 11.34208 | 5645.9 | 203.3373 |
| 5.0 | 770.4 | 5.184571 | 5564.367 | 57.17738 |
| 5.8 | 811.6333 | 5.579927 | 6051.467 | 78.65091 |
| 6.0 | 879.5667 | 5.018172 | 5483.267 | 79.74463 |
| 6.2 | 815.7 | 12.32841 | 6623.233 | 59.02377 |
| 6.4 | 1040.867 | 3.869001 | 7361.7 | 66.32357 |
| 6.6 | 881.6334 | 12.3051 | 7155.633 | 64.48636 |
| 6.8 | 931.0667 | 6.743064 | 8260.067 | 108.7139 |
| 7.0 | 879.5333 | 15.38667 | 9262.267 | 93.27428 |
| 7.2 | 944.0667 | 5.63577 | 9888.133 | 110.562 |
| 7.4 | 885.1334 | 4.994864 | 11663.63 | 115.7139 |
| 7.6 | 1110.833 | 10.33458 | 9953.067 | 92.87688 |
| 7.8 | 1055.667 | 11.23697 | 9687 | 113.8786 |
| 8.0 | 801.4667 | 6.630402 | 9300.633 | 133.8356 |
| 9.0 | 765.9 | 6.528398 | 8925.233 | 116.0779 |
| 10.0 | 861.7333 | 81.37076 | 9036.467 | 114.9727 |
| 11.0 | 850.0667 | 66.36089 | 9097.367 | 138.7611 |
| 12.0 | 860.4333 | 73.26695 | 9248.333 | 120.668 |

^a^average of 3 measurements

^b^standard deviation

Table S3. Fluorescence intensity response of compound **2** to selected analytes at three biological relevant pH values pH = 7.0, pH = 7.2 and pH = 7.4.

|  | 7.0 | | 7.2 | | 7.4 | |
| --- | --- | --- | --- | --- | --- | --- |
|  | Fluorescence intensity^a^ | SD^b^ | Fluorescence intensity^a^ | SD^b^ | Fluorescence intensity^a^ | SD^b^ |
| Na_2_S | 4095.348 | 9.731593 | 5922.502 | 2.186713 | 4942.137273 | 3.636001 |
| Blank | 2084.37 | 2.108321 | 1441.487 | 9.241722 | 849.77159 | 5.808594 |
| Cys | 500.6461 | 65.94364 | 1428.255 | 530.6954 | 1285.133357 | 6.503826 |
| GSH | 509.8861 | 15.18121 | 829.7701 | 282.4959 | 777.9897733 | 2.131563 |
| KSCN | 636.3148 | 0.053034 | 1403.161 | 0.620587 | 527.3614433 | 1.133614 |
| Na_2_SO_3_ | 317.0201 | 0.041 | 666.7984 | 4.38826 | 576.3203667 | 0.791498 |
| Na_2_SO_4_ | 828.0553 | 5.230812 | 1581.691 | 0.21375 | 644.7486133 | 1.039529 |
| Cys (500 equiv.) | 456.6578 | 6.283345 | 1771.401 | 7.747064 | 697.0977 | 3.645601 |
| GSH (500 equiv.) | 513.5861 | 1.458644 | 1015.174 | 1.854616 | 634.9619367 | 1.243023 |

^a^average of 3 measurements

^b^standard deviation





**Figure S8 |** (a) emission spectra of compound **2** excitation at 430 nm, (b) excitation spectra of compound **2** emission at 520 nm, (c) emission spectra of reduced form of compound **2** excitation at 430 nm, (d) excitation spectra of reduced form of compound **2** emission at 520 nm.

**

**

**Figure S9 |** Plot of the fluorescence intensity of compound **2** (10 μmol L^−1^) in the (a) concentration range of 0–20 μmol L^−1^ of H_2_S at 520 nm, (b) concentration range of 0–300 μmol L^−1^ of H_2_S at 520 nm.

**

**

**Figure S10 |** Determination of H_2_S concentration in spiked human serum sample using Na_2_S as internal standard (5–20 μmol L^−1^) by (a) fluorescent probe detection at 520 nm, (b) UV-Vis spectrophotometry method using methylene blue in spiked human plasma sample using Na_2_S as internal standard (5–20 μmol L^−1^) at 570 nm.
